# Synthesis of Lignin-based Phenol Terminated Hyperbranched Polymer

**DOI:** 10.3390/molecules24203717

**Published:** 2019-10-16

**Authors:** Lionel Longe, Gil Garnier, Kei Saito

**Affiliations:** 1 School of Chemistry, Bioresource Processing Research Institute of Australia (BioPRIA), Monash University, Clayton 3800, Australia; lionel.longe@monash.edu; 2 Department of Chemical Engineering, Bioresource Processing Research Institute of Australia (BioPRIA), Monash University Clayton 3800, Australia

**Keywords:** bio-based, hyperbranched, polyphenol, antioxidant

## Abstract

In this work, we proved the efficient synthesis of a bio-based hyper-branched polyphenol from a modified lignin degradation fragment. Protocatechuic acid was readily obtained from vanillin, a lignin degradation product, via alkaline conditions, and further polymerised to yield high molecular weight hyperbranched phenol terminated polyesters. Vanillic acid was also subjected to similar polymerisation conditions in order to compare polymerisation kinetics and differences between linear and hyperbranched polymers. Overall, protocatechuic acid was faster to polymerise and more thermostable with a degradation temperature well above linear vanillic acid polyester. Both polymers exhibited important radical scavenging activity (RSA) compared to commercial antioxidant and present tremendous potential for antioxidant applications.

## 1. Introduction

Hyperbranched polymers (HBP) have received increasing attention over the past two decades, as their field of the application keeps expanding every year [1,2]. The hyperbranched polymer structure is very similar to dendrimers with a high amount of end group, often used for post functionalisation or grafting reactions. However, HBP are often cheaper and easier to synthesise. For these reasons HBP have replaced dendrimers in many applications, and are now commonly used in various fields of application, including therapeutics, drug delivery, coatings, and as rheological or curing agents [3,4,5,6]. Recently, interest in the synthesis of the bio-based hyperbranched polymers has quickly grown as the logical consequence of the success of green chemistry in the polymer field [7,8,9,10,11,12,13,14]. However, to our knowledge, synthesis from lignin degradation products feedstock has yet to be reported.

Polyphenols are well known for their antioxidant properties. The synthesis of efficient bio-based polymers with radical scavenging properties has seen a growing interest in the past few years [15,16]. The most common strategy involves grafting a highly antioxidant molecule on a polymer backbone [17,18]. Phenol-terminated molecules, such as caffeic acid, are often investigated. To increase the phenol content, hyperbranched polymers and dendrimers are also starting to draw attention and should drastically improve the final radical scavenging activity [19,20,21,22,23].

In this work, we present the monomer preparation and synthesis of a phenol-terminated hyperbranched polymer. High antioxidant properties are expected, and radical scavenging activity will be monitored to determine the efficacy of the synthesised polymer. Biomass feedstock will be used for the monomer synthesis in order to create a bio-renewable polymer. Vanillin can be readily obtained from lignin degradation and already has well established in industrial production and commercial markets [24,25,26,27]. In strong caustic conditions, vanillin will yield protocatechuic acid (PA) in high yield and high purity. We report here the synthesis of a hyperbranched polymer by polycondensation of PA. Furthermore, similar conditions were employed with vanillic acid (VA) to compare final polymer properties.

## 2. Results and Discussion

**Figure 1:**
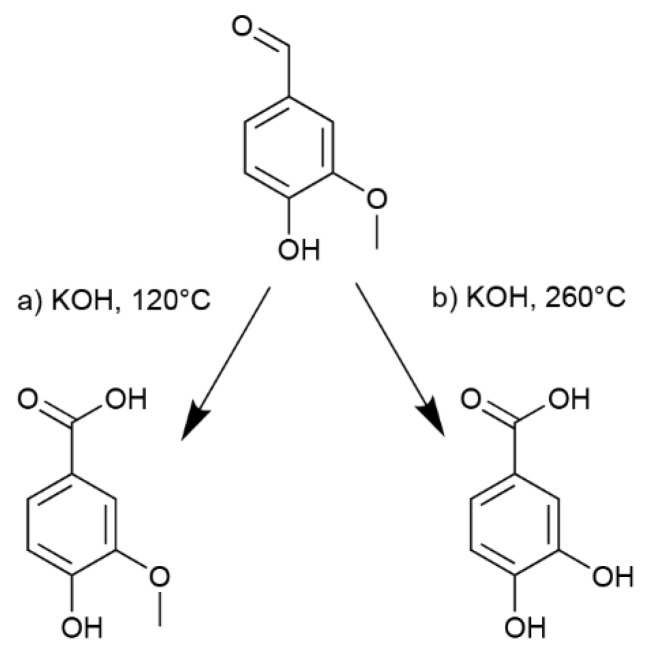
Synthesis of (**a**) vanillic acid (VA) and (**b**) protocatechuic acid (PA) from vanillin.

PA was readily obtained by caustic fusion of vanillin (Figure 1). Reaction temperature determines whether demethylation occurs in addition to the oxidation of the aldehyde group. With a temperature below 250 °C, vanillic acid (VA) is formed, while a higher temperature yields PA. Purity is highly dependent on the mixing method and homogeneous heating. The reaction is total, and protocatechuic acid and vanillic acid are obtained in high yield, 94% and 99%+ respectively.

**Figure 2:**
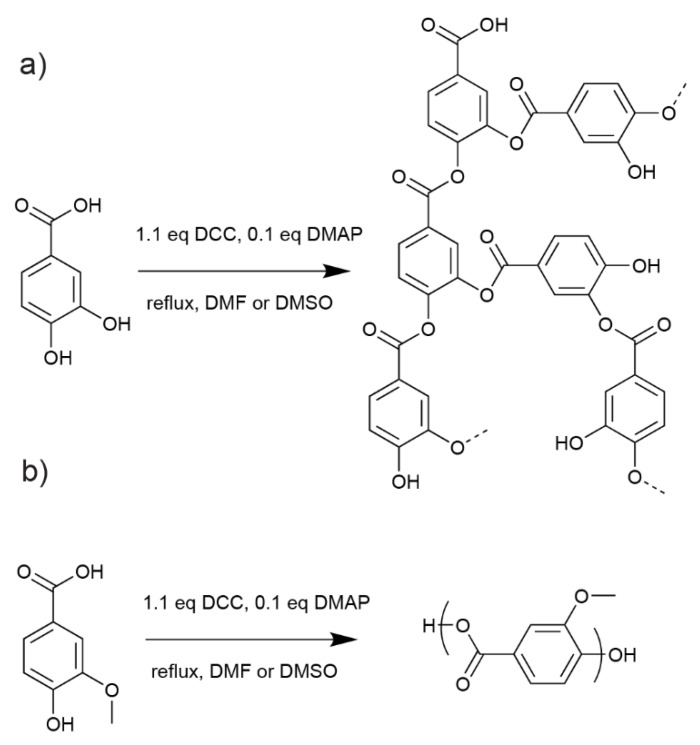
Polycondensation of (**a**) protocatechuic acid (PA) and (**b**) vanillic acid (VA) by Steglich esterification.

After isolation and purification, PA and VA were polymerised by polycondensation using Steglich esterification reaction in dimethyl formamide (DMF) (Figure 2). The Steglich esterification reaction is well known for the associated intensive work-up to remove dicyclohexylurea (DHU), a side product from the acid activation by *N*,*N*’-dicyclohexylcarbodiimide (DCC). In the present case, DCC was efficiently removed by Soxhlet extraction in acetone. Gel permeation chromatography (GPC) analysis only indicated a small difference between PA and VA-polymers. Overall, PA-polymer reached slightly higher molecular weight than VA and, correspondingly moderately faster polymerisation kinetics (Table 1). The presence of the methoxy group in VA is expected to reduce the phenol reactivity, due to steric hindrance and can be the cause of that difference. However, GPC equipment was calibrated versus linear polystyrene standards, and branched structures are known to reduce the accuracy of molecular weight determination [28]. Therefore, no major difference in polymerisation behaviour is to be noted at this stage.

**Figure 3:**
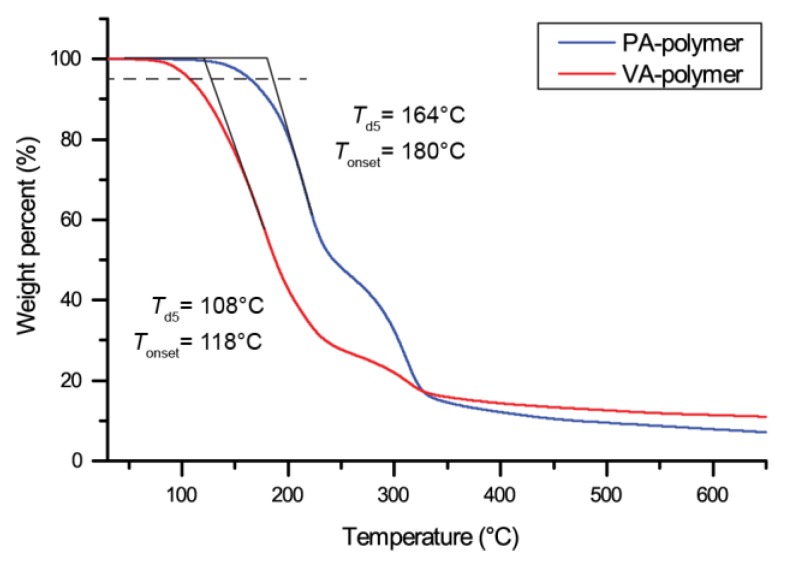
Thermogravimetric analysis of the decomposition of both PA and VA-based polymer.

The two polymers thermostability was compared by thermogravimetric analysis (TGA). A clear difference between the two 5% weight loss temperature (*T*_d5_) was observed, with PA-based polymer proving to be stable up to 164 °C, while VA-polymer started to deteriorate above 108 °C (Figure 3 and Table 1).

Analysis by differential scanning calorimetry (DSC) of PA and VA-polymer samples could not point out any clear glass transition temperature in the analysis range (−70 °C to 145 °C and 90 °C respectively), presumably due to the hyperbranched structure of PA-polymer and the low degree of polymerisation of VA-polymer.

**Figure 4:**
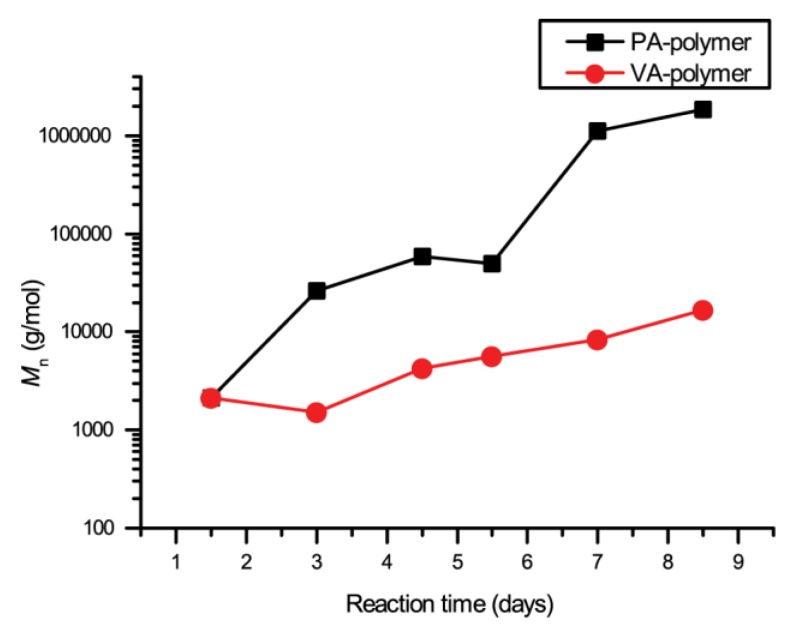
Evolution of polymers molecular weight during the polymerisation of protocatechuic acid and vanillic acid by Steglich esterification in DMSO, measured by gel permeation chromatography (GPC).

The overall increase in polymer molecular weight proved to be slow, possibly due to the low reactivity of phenols group. This issue can be partially circumvented by increasing the polymerisation temperature. High boiling point polar solvent proved to be best suited for this reaction. Polymerisation in dimethyl sulfoxide (DMSO) proved to be more efficient than DMF as it proved to be a better solvent for the polymer and allowed stronger heating. All other conditions were kept constant (Figure 2). Even in DMSO, the increase of the polymers molecular weight proved to be slow. Nevertheless, with long reaction time, ultra-high molecular weight polymers could be obtained (Figure 4). Samples with *M*_n_ of up to 2,000,000 g.mol^−1^ were successfully isolated. However, ^1^H NMR analysis of the synthesised polymer evidence a side reaction, due to interaction with the solvent (Figure S2). Moffat and Burdon previously reported that DCC and DMSO could react in the presence of acid catalyst—also known as Pfitzner-Moffatt conditions—and react with phenolic compounds [29]. Several compounds can be created from this reaction. The main reported behaviour was the alkylation of the phenols in the available *ortho* positions with thiomethoxymethyl groups. Binding of dicyclohexylurea to the molecules was also reported in the same position. Formation of cyclic hemithioacetal structures was also reported. In ^13^C NMR analysis, the signal in the δ = 25–50 ppm clearly indicated the binding of DHU molecules to the polymer structure.

The formation of methylthiomethyl esters and *N*-acylureas by the reaction of the carboxylic acid group has been reported in the literature [30] (Figure S1). However, considering the overall molecular weight of the final polymer sample, this reaction either (a) was not favourable or (b) was reversible in a way that does not prevent polycondensation. The latter case could, however, explain the overall slow kinetics of the polymerisation. Nevertheless, due to the high polymerisation degree, this reaction cannot be detected by NMR analysis.

**Figure 5:**
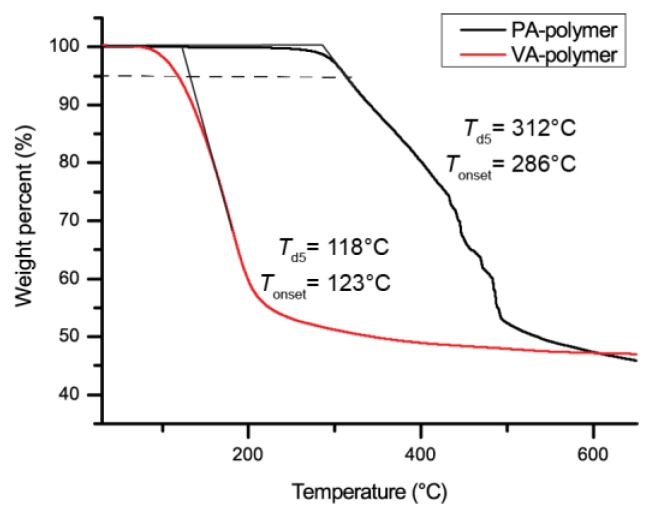
Thermogravimetric analysis of polymer from protocatechuic acid and vanillic acid.

The thermal stability of both polymers synthesised in DMSO was analysed by thermogravimetric analysis (Figure 5). Polymerisation in DMSO did not change the degradation temperature for VA-polymer, but remaining ash content greatly increased from 10% to 50%. Similarly, degradation of PA-polymer now also yields about 50% of the mass of ashes. The degradation temperature for PA-polymer, however, now greatly increased, and is now thermostable to up to 312 °C, probably partially, due to the 200-fold increase in molecular weight. During differential scanning calorimetry (DSC) analysis over a large range of temperatures (−70 °C to *T*_d5_ −20 °C) no clear glass transition temperature could be detected. Glass transition temperature of branched polymers can be hard to detect using DSC so other detection methods should be used in future work.

**Figure 6:**
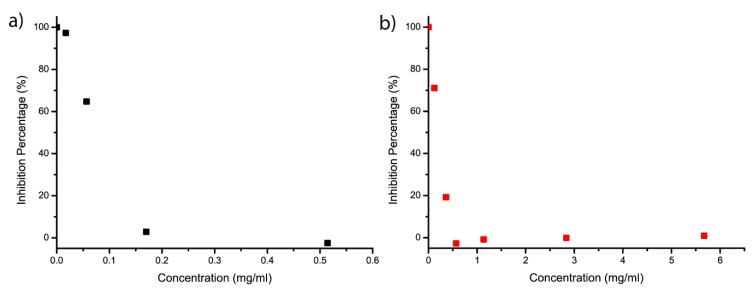
Inhibition percentage of (**a**) PA-polymer and (**b**) VA-polymer versus concentration. This was measured by bleaching of DPPH at 515 nm during radical scavenging assays.

Due to the presence of the phenolic group, antioxidant properties are expected from both polymers. The radical scavenging activity is measured by the determination of the amount of substrate that will bleach half the amount of a stable radical compound, 2,2-diphenyl-1-picrylhydrazyl (DPPH). The procedure is adapted from the recommendation of Kedare et al. [31] (Figure 6). As expected, on account of the higher amount of phenol group, due to its hyperbranched structure, PA-based polymer is almost twice as efficient as VA-polymer, with an EC_50_ (efficient concentration) of 0.08 mg/mol (Table 2). Antioxidant bio-based polymer represents a high interest in research and for industrial application. By grafting an increasing amount of phenol on the polymer backbone, the antiradical efficacy can be greatly improved (Table 2). Chitosan, a linear biopolymer with no phenol group is often studied as a polymer backbone for grafting antiradical compounds to create bio-based antioxidant polymers, and was compared here to linear VA-polymer. PA-polymer RSA was also compared to a bio-based polymer with similar structure (Figure S4). Chitosan grafted with molecules with multiple phenol group (caffeic acid), or dendritic structure (chitosan graft tannic acid, dendritic phenol) were selected for comparison. Additionally, RSA of two widely used industrial antioxidant (Irganox^®^ 1010 and Irganox^®^ 1098) are also reported and provide a benchmark for commercial antioxidant. Overall, our HBP have higher antiradical efficacy than all other biopolymers, but one, and is also two to four time more efficient than the commercial non bio-based options.

## 3. Materials and Methods

Chemicals and solvents: Chemicals were purchased from Sigma Aldrich (Sydney, NSW, Australia) (vanillin, 4-dimethylaminopyridine (DMAP), *N*,*N*’-Dicyclohexylcarbodiimide (DCC), lithium bromide), from Merck PTY (Melbourne, VIC, Australia) (Potassium hydroxide, anhydrous manganese sulphate) and from TCI (Tokyo, Japan) (deuterated dimethyl sulfoxide or DMSO) and used as received. Reagent grade solvents (anhydrous dimethylformamide or DMF, diethyl ether, hydrochloric acid 37% in water, Tetrahydrofuran or THF) were purchased from Thermo Fisher Scientific (Melbourne, VIC, Australia).

The molecular weight of polymers samples was analysed by size exclusion chromatography (SEC) in DMF or water. SEC in DMF was performed at 40 °C on a Tosoh EcosHLC-8320 equipped with double detectors (RI, UV 280 nm) using Tosoh alpha 4000 and 2000 columns. DMF/LiBr 0.1 M was used as a mobile phase (flow rate of 1 mL/min). Polystyrene standards were used for the calibration.

^1^H NMR and ^13^C qNMR experiments were performed in deuterated DMSO on a Bruker Avance 400 MHz NMR spectrometer equipped with a CryoProbe Prodigy 5 mm ^1^H/^1^H-^13^C/^15^N probe. NMR experiments were performed with the sample held at 25 ± 0.1 °C. Chemical shifts for all experiments are referenced using the Unified Scale relative to 0.3% tetramethylsilane in deuterated chloroform.

Radical scavenging activity was measured through the measurement of the bleaching of DPPH at 515 nm by UV-visible spectroscopy. A fresh solution of DPPH at 2.65 mg/mL in methanol is prepared and kept away from light in the fridge. The solutions of the substrate in DMSO are also prepared at a known concentration. In a test tube, 60 μL of the DPPH solution is added with a known volume of the substrate solution. The volume is then adjusted to 3 mL with DMSO. The test tube is then sealed closed and kept away from light to react for 3 h. After that time, the absorption of the solution at λ = 315 nm is measured. The absorption of the substrate alone is also measured to subtract its effect on the measurement. This is repeated as much as needed with different volumes of the substrate solution in order to gather data before and after the total quenching of DPPH. The residual absorption of totally quenched DPPH is also measured for more accurate results. Ascorbic acid was selected as standard, and its antiradical efficacy was also measured and compared to literature to confirm measurements (Figure S3, Table S1) [16]. UV measurements were performed on an Agilent Cary 60 UV-Vis cell reader spectrophotometer in quartz cells.

Degradation temperatures were measured by thermogravimetric analysis (TGA) performed on a Simultaneous Thermal Analyser (STA) 8000 from PerkinElmer. The heating rate was fixed to 30 °C/min under nitrogen flow.

Glass transition temperatures were measured by differential scanning calorimetry (DSC) on a DSC 8000 from PerkinElmer fitted with a dual-stage heat-exchanger cooling system Intracooler 2. Samples were analysed in aluminium pans and under nitrogen atmosphere. The heating rate was set to 10 °C/min and cooling rate 150 °C/min. Scanning ranges were set between −70 °C and up to the polymer degradation temperature, determined by TGA, minus 20 °C.

### 3.1. PA Synthesis

Protocatechuic acid was obtained by the caustic fusion of vanillin. In a nickel crucible potassium hydroxide (33 g, 0.588 mol) is added with a small volume of water (4 mL). A crucible is placed in an oil bath, and an overhead mechanical stirrer is installed. Heating is set to 260 °C and stirring is set to ca. 100 rpm. After a few minutes, the salt mixture becomes a homogeneous viscous mixture. Vanillin (12 g, 0.079 mol) is then carefully added into the solution. After 45 min, the reaction is stopped and allowed to cool down. The reaction mixture is dissolved in water (200 mL), and the product is then recovered by acidification/precipitation with hydrochloric acid. Filtration in a Buchner funnel yields 9.5 g of protocatechuic acid (0.061 mol). The aqueous phase is further extracted three times with diethyl ether (50 mL). The organic phase in then dried over anhydrous MgSO_4_ and evaporated under reduced pressure to recover an additional 2.1 g of protocatechuic acid (0.013 mol, 94% yield). ^1^H-NMR (400 MHz, *d*6-DMSO): δ 7.32 (s, H, ArH), δ 7.27 (d, 1H, ArH), δ 6.79 (d, 1H, ArH).

### 3.2. VA Synthesis

Synthesis of vanillic acid is achieved by an identical procedure, only maintaining reaction temperature at 150 °C. Potassium hydroxide (33 g, 0.588 mol) and water (4 mL) mixture are brought to a gel in the nickel crucible by maintaining heating at 150 °C and constant stirring ca. 100 rpm. Vanillin (12 g, 0.079 mol) is then added, and left to react for 45 min. After allowed to cool down, the mixture is dissolved in water (200 mL), and vanillic acid is crushed out by acidification with hydrochloric acid. 10.3 g of vanillic acid can be recovered (0.067 mol). Extraction of the aqueous phase with diethyl ether further yields 1.9 g of vanillic acid (0.012 mol, 99%+ yield). ^1^H-NMR (400 MHz, *d*6-DMSO): δ 7.44 (s, 1H, ArH), δ 7.43 (d, 1H, ArH), δ 6.84 (d, 1H, ArH), δ 3.92(s, 3H, CH_3_).

### 3.3. Polymerisation of VA and PA

Vanillic acid and protocatechuic acid are polymerised by polycondensation using Steglich esterification reaction. The phenol compound (3 mmol) is added to a 50 mL round bottom flask and dissolved in 30 mL of solvent (DMF or DMSO). 3.6 mg of DMAP (0.03 mmol) and 680 mg of DCC (3.3 mmol) are further added to the flask. Magnetic stirring is set up ca. 300 rpm until complete homogenisation of the solution. The round bottom flask is then fitted with a condenser, and the reaction is allowed to carry on in reflux condition. After the reaction, the mixture is left to cool down and is then filtered to remove insoluble side product dicyclohexylurea. Complete removal of DHU is reached by overnight Soxhlet extraction with acetone. The soluble fraction is then dissolved in DMSO and added dropwise to a large volume of THF to crush out high molecular weight fractions.

## 4. Conclusions

In this work, we reported the synthesis and polymerisation of a bio-based monomer from lignin, PA. Its AB_2_ configuration was compared to another monomer with simple AB structure, VA. During the polymerisation study side reaction with the solvent was detected. However, in those conditions, the final synthesised polymer gained better thermal properties and was able to reach a high degree of polymerisation. Both polymers exhibited high antioxidant properties, due to the presence of phenolic groups in the structure. NMR spectroscopy was used to characterise the different side reactions and their effect on the polymer structure. In future work, we ought to improve the PA-polymer characterisation, focussing on the structure (degree of branching) and physico-mechanical properties. This polymer is expected to have a great impact as a 100% bio-based polymer with antioxidant properties. Applications are expected for paint, packaging, emulsion, formulation or drug delivery application. In addition, the phenol terminated structure allows easy post-modification and will be considered in future work to create further applications for this polymer.

## Figures and Tables

**Table 1:** Physical properties of polymers from protocatechuic acid and vanillic acid.

	*M*_n_ (g/mol)	*Đ*	*T*_onset_	*T*_d5_
PA-based polymer	7900	3.1	180 °C	164 °C
VA-based polymer	6400	2.6	118 °C	108 °C

**Table 2:** Antioxidant properties of synthesised polymers compared to literature and commercially available antioxidant.

	EC_50_ (mg/mL)	Antiradical Efficacy (mmol) ^a^
PA-polymer	0.08	0.76
VA-polymer	0.22	0.30
Chitosan	/	n.d. [17]
Chitosan graft caffeic acid	/	0.16 [17]
Chitosan graft tannic acid	/	5.8 [18]
Dendritic phenol	/	0.36 [19]
Irganox^®^ 1010	/	0.18 [32]
Irganox^®^ 1098	/	0.36 [19]

^a^: DPPH equivalents per gram of material; n.d.: not detected.

## Supplementary Materials

The following are available online: DPPH analysis of ascorbic acid standard and NMRs of the synthesised polymer are available in supporting information.
