# Supplementary material for: Synthesis of Lignin-based Phenol Terminated Hyperbranched Polymer
Source: Molecules. 2019 Oct 16;24(20):3717. doi: 10.3390/molecules24203717 (PMC6832395; doi:10.3390/molecules24203717)
Supplement: Supplementary file 1 [file molecules-24-03717-s001.pdf]

molecules

Supportiing Information

Synthesis of lignin-based phenol terminated hyperbranched polymer

Lionel Longe [1], Gil Garnier [2,*] and Kei Saito [1,*]

[1] Monash University, School of Chemistry, Bioresource Processing Research Institute of Australia (BioPRIA), Clayton 3800, Australia
[2] Monash University, Department of Chemical Engineering, Bioresource Processing Research Institute of Australia (BioPRIA) Clayton 3800, Australia
* Correspondence: kei.saito@monash.edu ; gil.garnier@monash.edu

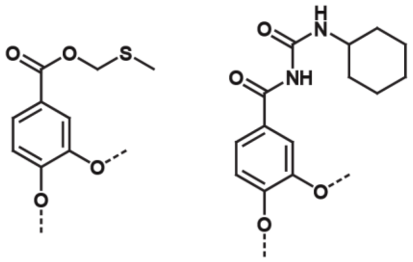

Figure S2: Carboxylic acid can react with activated DMSO or bind with DHU to form methylthiomethyl ester (left) or *N*-acylurea (right) structures

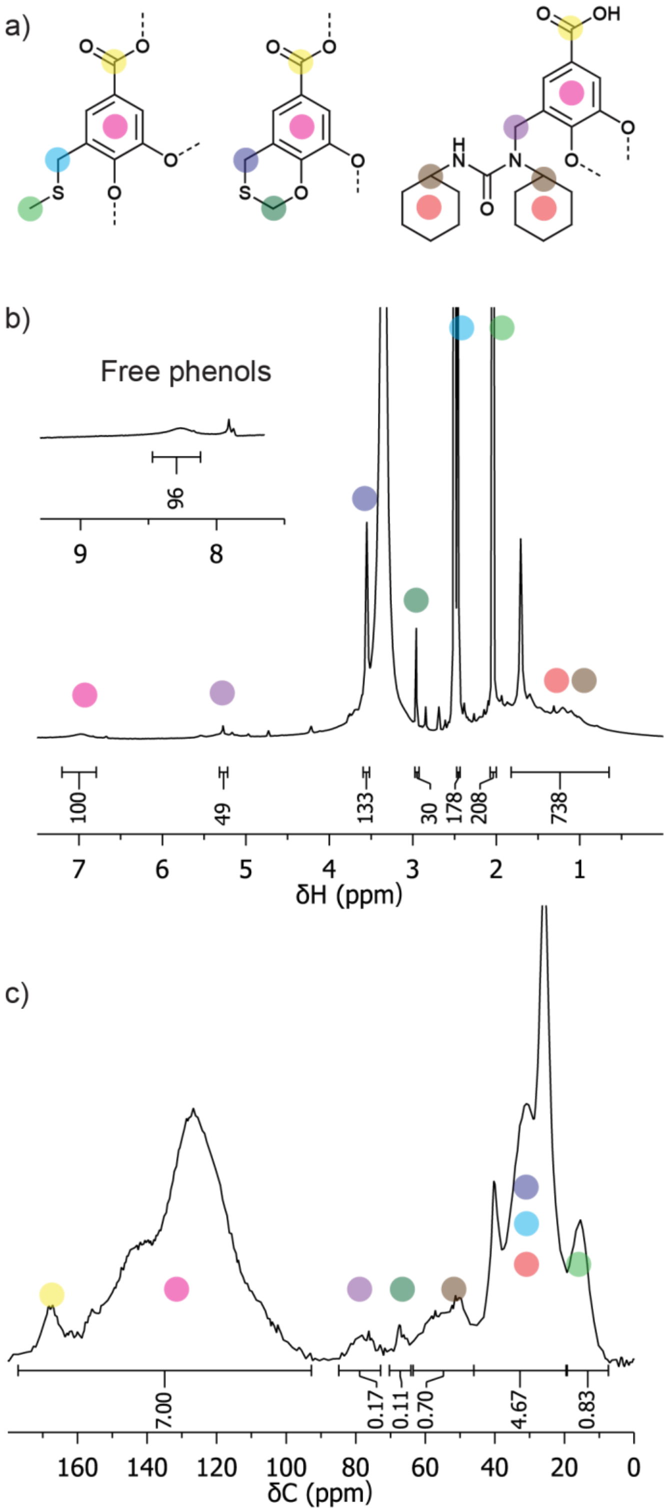

Figure S1: a) Three different structures that can arise from DMSO activation by DCC and reaction with PA b) 1H NMR of PA-polymer highlighting evidence of side reactions in d6-DMSO c) 13C NMR of PA-polymer in d6-DMSO

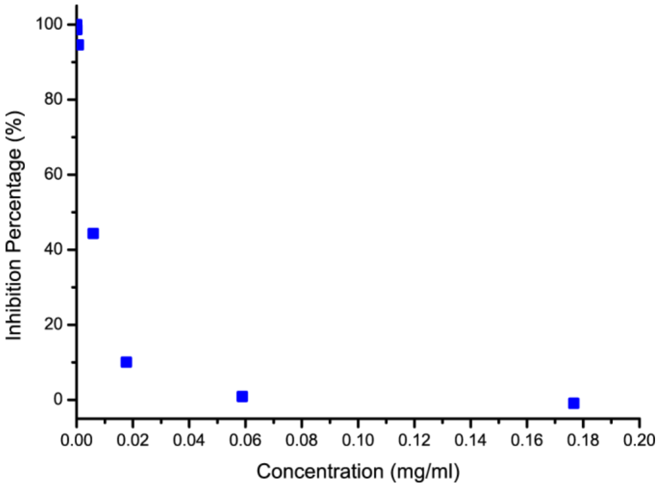

Figure S3: Inhibition percentage of ascorbic acid versus concentration. Measured by bleaching of DPPH at 515 nm during radical scavenging assays[1]

Table S1: Antiradical efficacy of ascorbic acid compared to literature

	EC_{50} (mg/ml)	Antiradical efficacy (mmol) [a]
ascorbic acid	0.0052	12.7
ascorbic acid [1]	/	12.0

[a]: DPPH equivalents per gram of material

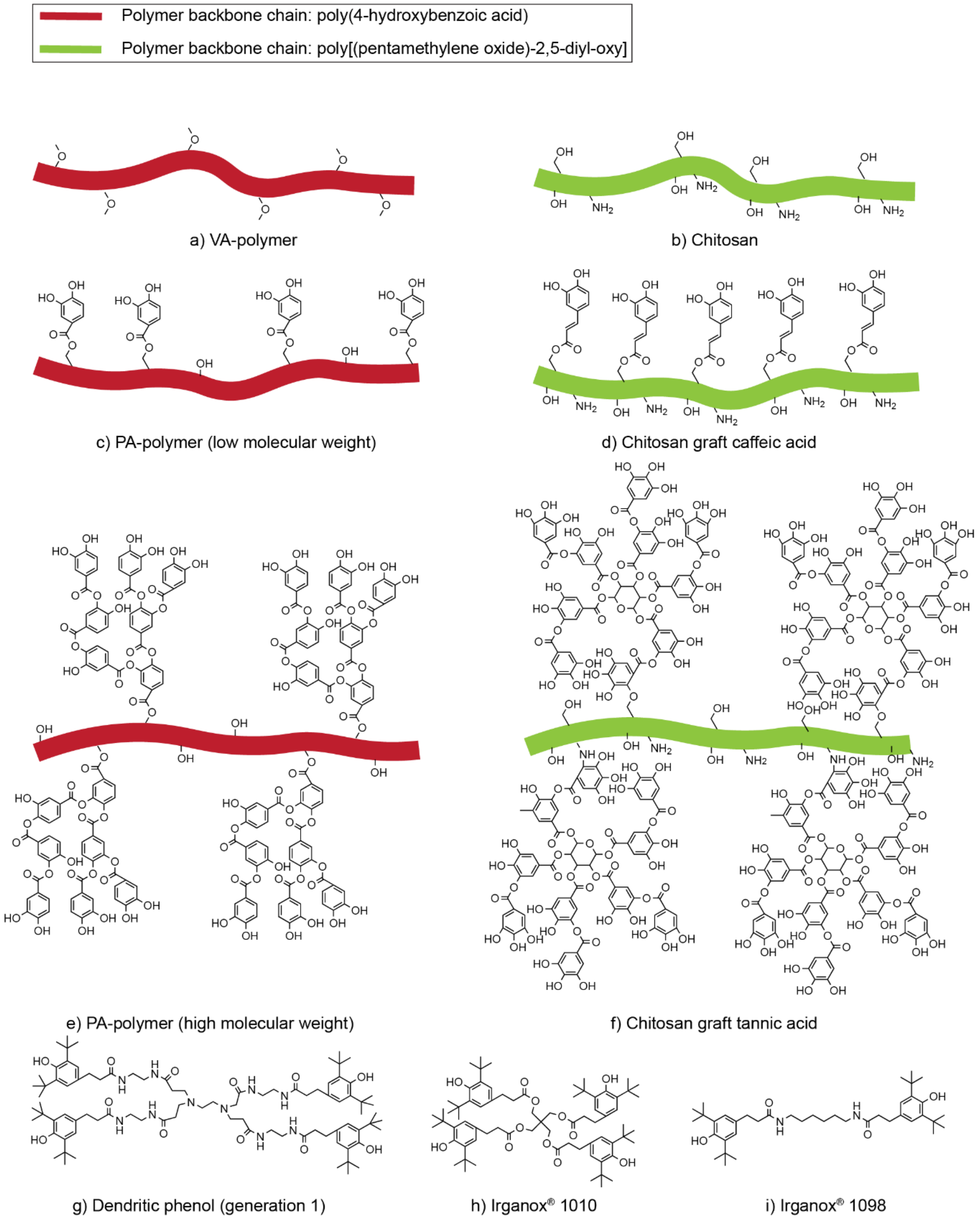

Figure S4: Structure of synthesised polymers (a, c, e) and literature (b, c, f, g) and commercially available (h, i) antioxidant.

References

1. Omidi, S., Kakanejadifard, A. Modification of chitosan and chitosan nanoparticle by long chain pyridinium compounds: Synthesis, characterization, antibacterial, and antioxidant activities. *Carbohydr. polym*., **2019** *15*; 477-485

© 2019 by the authors. Submitted for possible open access publication under the terms and conditions of the Creative Commons Attribution (CC BY) license (http://creativecommons.org/licenses/by/4.0/).
